# Palliative long-term abdominal drains versus repeated drainage in individuals with untreatable ascites due to advanced cirrhosis: study protocol for a feasibility randomised controlled trial

**DOI:** 10.1186/s13063-018-2779-0

**Published:** 2018-07-27

**Authors:** Lucia Macken, Louise Mason, Catherine Evans, Heather Gage, Jake Jordan, Mark Austin, Nick Parnell, Max Cooper, Shani Steer, Justine Boles, Stephen Bremner, Debbie Lambert, David Crook, Gemma Earl, Jean Timeyin, Sumita Verma

**Affiliations:** 1 Department of Clinical and Experimental Medicine, Brighton and Sussex Medical School, Main Teaching Building, North South Road, University of Sussex, Falmer, Brighton, East Sussex BN1 9PX UK; 2 Department of Gastroenterology and Hepatology, Brighton and Sussex University Hospitals Trust, Royal Sussex County Hospital, Eastern Rd, Brighton, East Sussex BN2 5BE UK; 3 Department of Palliative Medicine, Brighton and Sussex University Hospitals Trust, Royal Sussex County Hospital, Eastern Rd, Brighton, East Sussex BN2 5BE UK; 4 King’s College, Cicely Saunders Institute, Department of Palliative Care, Policy and Rehabilitation, Bessemer Road, London, SE5 9PJ UK; 5 Sussex Community NHS Foundation Trust, Brighton General Hospital, Elm Grove, Brighton, BN2 3EW UK; 6 Surrey Health Economics Centre, School of Economics, Faculty of Arts and Social Sciences, University of Surrey, Guildford, Surrey GU2 7XH UK; 7 Brighton & Sussex Clinical Trials Unit, Room 204 Bevendean House, University of Brighton, Falmer, BN1 9PH UK; 8 Department of Primary Care and Public Health, Brighton and Sussex Medical School, Mayfield House, Brighton, BN1 9PH UK

**Keywords:** Ascitic fluid, End-stage liver disease, Paracentesis, Permanent indwelling peritoneal catheter, Palliative care, Healthrelated quality of life, Quality-adjusted life years, Healthcare economics

## Abstract

**Background:**

UK deaths due to chronic liver diseases such as cirrhosis have quadrupled over the last 40 years, making this condition now the third most common cause of premature death. Most patients with advanced cirrhosis (end-stage liver disease [ESLD]) develop ascites. This is often managed with diuretics, but if refractory, then the fluid is drained from the peritoneal cavity every 10–14 days by large volume paracentesis (LVP), a procedure requiring hospital admissions. As the life expectancy of patients with ESLD and refractory ascites (if ineligible for liver transplantation) is on average ≤ 6 months, frequent hospital visits are inappropriate from a palliative perspective. One alternative is long-term abdominal drains (LTADs), used successfully in patients whose ascites is due to malignancy. Although inserted in hospital, these drains allow ascites management outside of a hospital setting. LTADs have not been formally evaluated in patients with refractory ascites due to ESLD.

**Methods/design:**

Due to uncertainty about appropriate outcome measures and whether patients with ESLD would wish or be able to participate in a study, a feasibility randomised controlled trial (RCT) was designed. Patients were consulted on trial design. We plan to recruit 48 patients with refractory ascites and randomise them (1:1) to either (1) LTAD or (2) current standard of care (LVP) for 12 weeks. Outcomes of interest include acceptability of the LTAD to patients, carers and healthcare professionals as well as recruitment and retention rates. The Integrated Palliative care Outcome Scale, the Short Form Liver Disease Quality of Life questionnaire, the EuroQol 5 dimensions instrument and carer-reported (Zarit Burden Interview) outcomes will also be assessed. Preliminary data on cost-effectiveness will be collected, and patients and healthcare professionals will be interviewed about their experience of the trial with a view to identifying barriers to recruitment.

**Discussion:**

LTADs could potentially improve end-of-life care in patients with refractory ascites due to ESLD by improving symptom control, reducing hospital visits and enabling some self-management. Our trial is designed to see if such patients can be recruited, as well as to inform the design of a subsequent definitive trial.

**Trial registration:**

ISRCTN, ISRCTN30697116. Registered on 7 October 2015.

**Electronic supplementary material:**

The online version of this article (10.1186/s13063-018-2779-0) contains supplementary material, which is available to authorized users.

## Background

UK deaths due to chronic liver disease such as cirrhosis have quadrupled over the last 40 years, making this condition the third most common cause of premature death [1]. Ascites — an abnormal accumulation of fluid in the peritoneal cavity — is present in up to 90% of patients with advanced cirrhosis [2, 3], resulting in frequent hospitalisations due to debilitating episodes of pain and breathlessness. In the early stages, this condition can be managed using diuretic therapy, but as the condition progresses (end-stage liver disease [ESLD]) the ascites becomes unresponsive to medical treatment. In the absence of liver transplantation, a diagnosis of refractory ascites confers a median life expectancy of ≤6 months [3–5].

End-of-life care in patients with ESLD and refractory ascites has not been a research priority. More than 70% of patients with ESLD die in hospital [6], a figure substantially — and in our view unacceptably — higher than that of 40% for patients with terminal cancer [7]. The most common palliative management for refractory ascites due to ESLD is large volume paracentesis (LVP), performed every 10–14 days [3]. This involves a costly 24–48 h hospital admission, insertion of a temporary abdominal drain and removal of up to 15 L of ascitic fluid over 4–6 h. There is simultaneous administration of intravenous 4.5% or 20% (w/v) human albumin solution, 8–10 g per 1 L of ascitic fluid removed [3]. Consequently, patients often delay the hospital visits until their ascites is tense and painful [8], thus reducing their quality of life (QOL) [9]. Individuals with refractory ascites often have contraindications to alternative invasive procedures such as the transjugular intrahepatic portosystemic shunt (TIPS) [10] and/or the automated low-flow ascites (ALFA) pump [11].

In this feasibility randomised controlled trial (RCT) we will investigate the use of a simple and less invasive device, the long-term abdominal drain (LTAD), in patients with refractory ascites due to ESLD. This technique involves placing a tunnelled drain through the abdominal wall, with ultrasound guidance, and with the patient under local anaesthetic. Once the drain is in place, the patient’s ascites can be drained in the patient’s usual place of residence. Community nurses or (where willing) carers can then remove smaller volumes (1–2 L) of ascitic fluid in about 5–10 min, usually two to three times a week dependent on patient preference. These devices have been extensively utilised in patients whose ascites is due to advanced malignancy, and they have been shown to be both clinically effective and cost-effective. They have low complication rates and offer improved QOL [12, 13] (Mullan D, Laasch H-U, Jacob AHH:Tunnelled intraperitoneal catheters in the management of malignant ascites: complications and cost implications [2012], unpublished). In terms of palliative care in refractory ascites due to ESLD, there may be additional benefits, including the involvement of patients and carers in the management of this condition; reduced complications through the regular removal of smaller volumes of fluid; and, importantly, reduced stigma associated with hospital-based LVP (voiced to us as a concern by service users).

Given the relative success of these devices in patients with ascites due to malignancy, there is a clear need for an RCT in ESLD and refractory ascites comparing the use of palliative LTADs to current standard of care (LVP), with end-of-life QOL and cost-effectiveness as major outcomes. LTADs have not been specifically assessed in patients with ESLD because of the potentially increased risks of bleeding (due to coagulopathy) [14] and infections, specifically spontaneous bacterial peritonitis [15, 16].

There are additional concerns related to this specific patient group. We do not know whether such individuals would be able or even willing to participate in an RCT, given their potentially higher prevalence of alcohol and substance misuse and other psychosocial issues. Similarly, there is uncertainty as to the most appropriate assessment tools and outcome measures. Finally, given the complex end-of-life care needs of this cohort, concerns remain to patients, carers and healthcare professionals (HCPs) over a strategy that moves care away from the hospital to the community. To address these issues we have designed a feasibility study to inform the development of a subsequent definitive RCT.

## Methods/design

### Aim, design and setting of the study

The aim of this study is to assess the feasibility of conducting a future RCT of the safety, clinical effectiveness and cost-effectiveness of refractory ascites management using the LTAD against current standard of care (LVP) in patients with ESLD when liver transplant is not an option. This document is based on v6.0 of the protocol (13 October 2017). This multicentre trial has been designed in accordance with phase 2 of the Medical Research Council (MRC) Complex Interventions Framework [17] and the Method Of Researching End of Life Care guidance (MORECare) [18].

This feasibility RCT is being conducted both at a hospital (the Royal Sussex County Hospital [RSCH], Brighton, Sussex, UK and the Princess Royal Hospital [PRH], Hayward’s Heath, Sussex, UK, both part of the Brighton and Sussex University Hospital [BSUH] National Health Service [NHS] Trust; Worthing Hospital, Worthing, Sussex, UK [Western Sussex NHS Foundation Trust], Plymouth Hospitals NHS Trust, Plymouth, UK, Blackpool Victoria Hospital, Blackpool, UK [Blackpool Hospitals NHS Foundation Trust] and Southampton General Hospital, Southampton, UK [University Hospital Southampton NHS Foundation Trust]) and in a community setting (The Sussex Community Trust). Those randomised to the LTAD will be followed up in the community. This 3-year study is planned to run from September 2015 until September 2018.

### Characteristics of participants

Patients will be identified from acute medical units, outpatients and gastroenterology and hepatology wards. They will be approached for the study at the participating centres by the research team after having been identified by the local medical team as being potentially eligible.

### Inclusion criteria

The inclusion criteria are as follows:

1. Age ≥ 18 years, with no upper age limit
2. Untreatable (refractory) ascites defined as:
  - Ascites that is unresponsive to fluid and sodium restriction and high-dose diuretic treatment (spironolactone 400 mg/day and/or furosemide 160 mg/day) and/or intolerance of diuretics [19, 20]
  - Ascites that recurs rapidly after LVP (requiring one or more LVP/month).
3. Child-Pugh score [21] of ≥ 9 unless specifically decided by the medical team that they are to receive only palliative treatment
4. Registered with a general practitioner (GP) in the Community Trusts serving the participating centres
5. Ability to speak, read and understand English
6. Capacity to give written informed consent as assessed by using a Capacity to Consent Checklist (see Additional file 1)

### Exclusion criteria

The exclusion criteria are as follows:

1. Loculated or chylous ascites
2. Presence of > grade 1 hepatic encephalopathy (specified by West Haven Criteria [22])
3. Evidence of active infection, which in the Investigator’s opinion would preclude insertion of an LTAD, e.g. bacterial peritonitis. Such patients could be reconsidered for inclusion in the trial if infection has been successfully treated
4. Eligible for liver transplantation, in the opinion of the hepatology multidisciplinary team (MDT) and according to national guidelines [23]
5. Psychosocial issues which, in the medical team’s opinion, would preclude engagement with the trial, such as posing a risk to the safety of oneself or the research team

As this is a feasibility study, we will not specifically exclude patients based on abnormal haemostasis measurements. Consistent with local practice, those individuals with a platelet count of < 50 × 10^9^ and/or an international normalised ratio (INR) of > 1.7 will be given blood and/or clotting products prior to receiving an LTAD or LVP.

Potential participants can be considered for inclusion in this trial even if they are currently participating in another research study, as long as their medical team are confident that participation in the current trial would be logistically feasible and not unduly onerous for the participants.

While we would prefer that potential carers/consultees are identified for each participant, their absence will not preclude study participation.

### Consent

Suitable participants will be identified by the usual medical teams. A research team member (to include Chief Investigator [CI], co-investigators, Principal Investigators [PIs], nurse and research fellow) will provide patient information sheets (PISs) to potential research participants and give an explanation about the study including ascites management. Patients will be provided at least 48 h to read the PIS. If willing, consent will be obtained in hospital by a research team member. If the research team is the usual medical team, to avoid any potential conflict of interest, potential participants will be discussed at the weekly liver multidisciplinary meeting (MDM). Capacity to give informed consent will be carefully assessed (see inclusion criteria). In the event that capacity is lost during the trial, the participant’s nominated consultee will be approached to determine whether the participant should continue in the study. If a consultee has not been nominated or is unavailable, then the participant’s usual medical consultant (independent from the research team) will be consulted to decide whether it is in the participant’s best interests to continue in the study.

### Randomisation

Patients who fulfil the inclusion and exclusion criteria and give written informed consent to participate in this trial will be randomised on a 1:1 basis to either Group 1: LTAD or Group 2: LVP (current standard of care). The allocations will be made by minimising on (1) centre, (2) Child-Pugh score and (3) gender. No stratification will be utilised. Minimisation will be implemented using an independent system hosted at King’s Clinical Trials Unit (KCTU). Patients will be enrolled by the research team member, who will log into the web-based system and enter patient ID number, recruiting site, gender and Child-Pugh score. The system will automatically generate a confirmation email informing the research team of the outcome of allocation.

Patients will be followed up for 12 weeks. With the participants’ agreement we will inform their GPs about their participation in the trial.

### Interventions

#### Group 1: LTAD

**Fig. 1:**
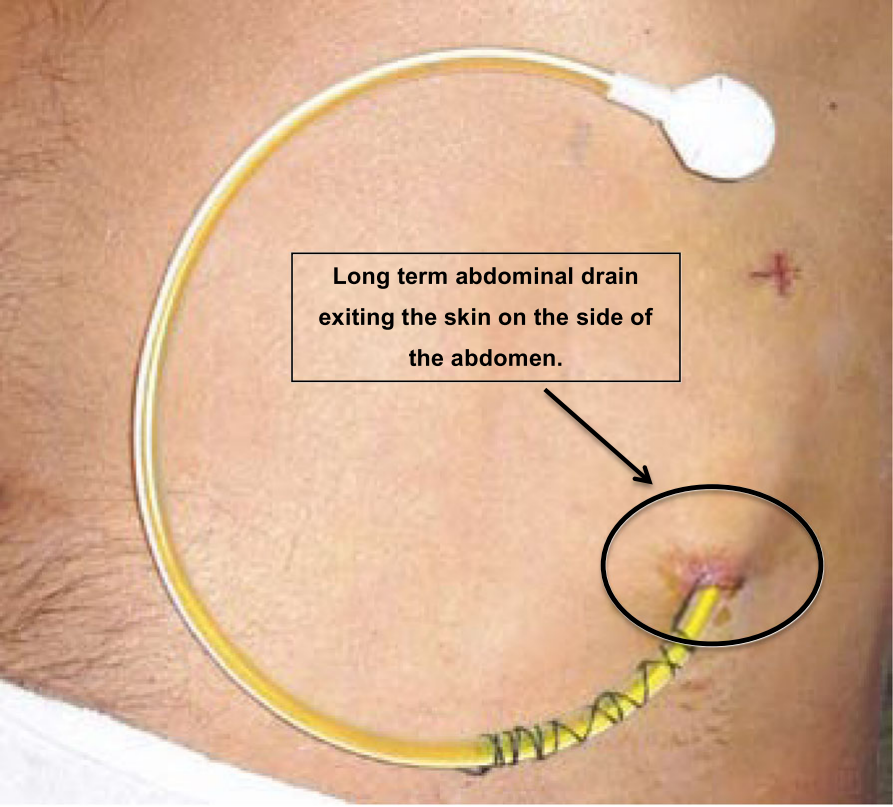
Rocket Medical Long-Term Abdominal Drain in situ [25]

There are two LTADs currently available in the UK: the PleurX™ (UK Medical Ltd., Basingstoke, UK [24]) and the Rocket® (Rocket Medical, Watford, UK [25]). We have chosen to use the Rocket device (Fig. 1) [25] primarily because our local clinicians and community nursing teams are already familiar with it. Rocket Medical already has an established training and support programme for local community nurses and care homes. In addition, our earlier experience [26] suggests that the Rocket devices are easier to insert than the PleurX™ devices and that they require less expensive consumables that can currently be prescribed by community practitioners.

#### Procedure for insertion of LTAD

Insertion of the LTAD will be performed in hospital in a side room, using bedside ultrasound guidance. Insertion will only be performed if, within the week leading up to planned LTAD insertion, haemostatic function (including INR and platelet count) has been checked and blood products administered as necessary. Where INR is > 1.7, patients will receive up to two volumes of fresh frozen plasma (FFP), transfused according to patient weight and INR, immediately prior to drain insertion. Where the platelet count is ≤ 50 × 10^9^, patients will be given one to two pools of platelets immediately prior to insertion of the drain.

To ensure consistency, it would be ideal that all LTADs are inserted at one site (RSCH), but if this is not possible due to patient preference or logistic issues, they will be inserted at local sites, usually by an interventional radiologist.

The Rocket® LTAD will be inserted using a combination of tunnelled and Seldinger technique as stated in the Rocket® information sheet [25]. After confirming the location of the insertion site using bedside ultrasound and skin preparation with Chloraprep™ (chlorhexidine gluconate and isopropyl alcohol), a local anaesthetic (up to 10 ml 1% or 2% lidocaine) will be administered at the incision site and along the proposed tunnel tract. A small incision is made where the catheter will enter the abdominal cavity. The introducer needle will be inserted through the incision into the peritoneal cavity and a guide wire is passed through the needle, which will then be removed. A second incision (exit site) will be made approximately 5 cm medial from the first, where the catheter will exit the tunnel. The catheter will be tunnelled from exit site incision to the first incision site with the tunneller, making sure that the cuff is midway between the first and second incision sites. A split-sheath dilator will then be passed over the guide wire, and the inner dilator and guide wire removed, leaving the split sheath in situ.

The tunneller is then removed from the catheter, which is then passed through the split sheath, separating the split sheath and ensuring that all of the catheter is contained within the peritoneum. The last piece of the split sheath is then removed. The catheter is then adjusted along the tunnel, so the cuff moves towards the exit site, ensuring that any kinks are removed from the catheter. Finally, both incision sites will be sutured (avoiding the catheter) and a dressing applied.

Participants will receive antibiotic prophylaxis (ciprofloxacin 500 mg/day) or an equivalent antibiotic (if there is a contraindication to ciprofloxacin), dependent on local practice.

We will provide guidance to the participant and carer (where present) on how to use the LTAD, based on the information previously supplied in the PIS. Participants will also be given an information sheet provided by Rocket Medical [25]. Participants will be referred to their community nursing service. A Rocket Medical discharge letter will be sent to their GPs and the community nursing team. Rocket Medical will also be informed so that they can organise any further support/training for patients, carers and community nurses. In addition, we will arrange for drainage bags to be delivered directly to the participant’s usual place of residence on request by the community nurses.

The community nurses will visit the participants in their homes and either perform the drainage procedure themselves or supervise the drainage of ascites. The frequency of these visits will depend on the participant’s ascites-related symptoms, but work in ascites due to malignancy [12, 27] indicates that two to three visits each week are most commonly required, with approximately 1–2 L of ascites being drained each time. It is recommended that the drainage frequency not exceed three times per week. In the event that participants and/or carers wish to perform self-drainage, they will be trained to do so by the community nurse.

The Integrated Primary Care Team (IPCT) will closely monitor trial participants allocated to the LTAD arm. We expect that this will happen two to three times a week if the community nurses are performing ascitic fluid drainage.

For participants who live in a care home or move to a care home (with or without nursing), the follow-up procedure would be the same as for patients who live at home. In such cases we would seek approval from the care home managers. For those requiring hospice care, this would be a temporary stay, since hospices do not generally provide long-term care. Again, permission will be sought from the hospice team to visit the participants for follow-up and only if such visits remain acceptable to the participants.

#### Group 2 standard of care (LVP)

Participants randomised to LVP [3], the current standard of care, will be admitted to hospital as either a self-referral or via their GP, whichever is current local practice. They will undergo LVP as clinically indicated. LVP involves the insertion of a peritoneal drain for up to 6 h and removal of up to 15 L of ascites. If the total volume of fluid to be removed is > 5 L, intravenous 4.5% or 20% (w/v) human albumin solution, 8–10 g per 1 L of ascitic fluid removed, will be administered [3].

As with Group 1, participants will receive antibiotic prophylaxis (ciprofloxacin 500 mg once a day or an equivalent antibiotic (if there is any contraindication to ciprofloxacin), dependent on local practice).

For both groups there will be two weekly visits with a research team member for questionnaire-based and clinical assessments as well as routine clinical blood samples (as discussed in subsequent sections). We anticipate that these two weekly contacts will improve adherence to the protocol.

### Clinical follow-up

While participating in this trial, for no individual will routine clinical care be modified or denied whether in the community, primary care or hospital setting. This will include symptomatic relief for pain (including use of opioids), shortness of breath, confusion (hepatic encephalopathy), jaundice or itching. Use of diuretics will be permitted in both groups. As is the current standard of care in patients with ESLD, the use of certain drugs (e.g. non-steroidal anti-inflammatory drugs, aminoglycosides) will be contraindicated [3].

Palliative care needs and concerns will be reassessed at each visit for each participant using the Integrated Palliative care Outcome Scale (IPOS) questionnaire [28] (see subsequent sections). If a high level of specialist palliative care need is identified (as defined within a distress protocol standard operating procedure [SOP]), through the IPOS questionnaire, a research team mini MDM (either face-to-face or virtual) will be convened to agree on the most appropriate way forward. As is standard clinical practice, referrals to a specialist service by the usual healthcare providers can also occur irrespective of any trial assessments or advice. If that occurs, consistent with standard practice, a referral is simultaneously made to a community (district) nurse, if this was not already done for another reason.

It may become necessary, after discussion with the CI and the Trial Management Group (TMG), to remove the LTAD. Reasons for this could include (1) patient request, (2) serious adverse reaction (SAR) assessed by the CI as being directly related to the LTAD and (3) significant deviation from the study protocol with potential for harm (for example the participant not allowing community nurses to enter residence to perform drainage).

The contact telephone numbers for key members of the research team will be provided to the participants. Out of hours, participants will be encouraged to contact their GP or attend the accident and emergency department of their local hospital, as per usual standard of care.

### Outcome measures

The objectives of this feasibility study therefore are to explore:

- Properties of different outcome measures (specifically health resource utilisation and QOL instruments) to ascertain the most appropriate primary outcome for the full trial and use the chosen primary outcome measure to inform sample size calculations from estimates of the standard deviations, for the full trial
- Resource implications of the LTAD compared to standard of care (LVP), including a preliminary assessment of cost-effectiveness to indicate whether a full trial is worthwhile
- The number of eligible patients
- The extent of HCP support in identifying possible participants
- Symptom burden in patients with ESLD and refractory ascites
- Informal carer/family perceived burden (if appropriate)
- Whether patients are willing to be randomised to LTAD, rather than LVP
- Acceptability of and adherence to home ascites drainage
- Attrition rates, including attrition due to death, illness or other causes
- Complication rates inclduing peritonitis
- Willingness to participate in a qualitative interview (optional)
- Acceptability of the LTAD to patients, carers and clinical staff using qualitative methods (optional)
- Acceptability of questionnaires

We will therefore collect data on a range of candidate primary outcome measures, including QOL and health resource utilisation. The primary outcome measure(s) for the definitive trial will be decided by the research team, including service users, on review of the final analysis of this feasibility study.

### Study success criteria

The study success will be based on the following criteria:

- The percentage of study period time in hospital for the LTAD group is < 50% of that for the LVP group (where the study period time is the number of days from the date of LTAD insertion to the end of the study period or the patient’s death (whichever is earliest); time spent in hospital is the number of bed days used).
- The attrition rate is not > 50%.
- There is < 10% overall rate of LTAD removal due to one or more of the following complications: peritonitis, failed insertion, bleeding and blockage.
- Each patient has completed 80% of the questionnaires and qualitative interviews.

### Data collection

Data will be collected on an electronic case report form (eCRF), using the MACRO electronic data capture system provided by KCTU and hosted on the King’s College London (KCL) server. The system is compliant with Good Clinical Practice (GCP), with a full audit trail and formal database lock functionality.

**Fig. 2:**
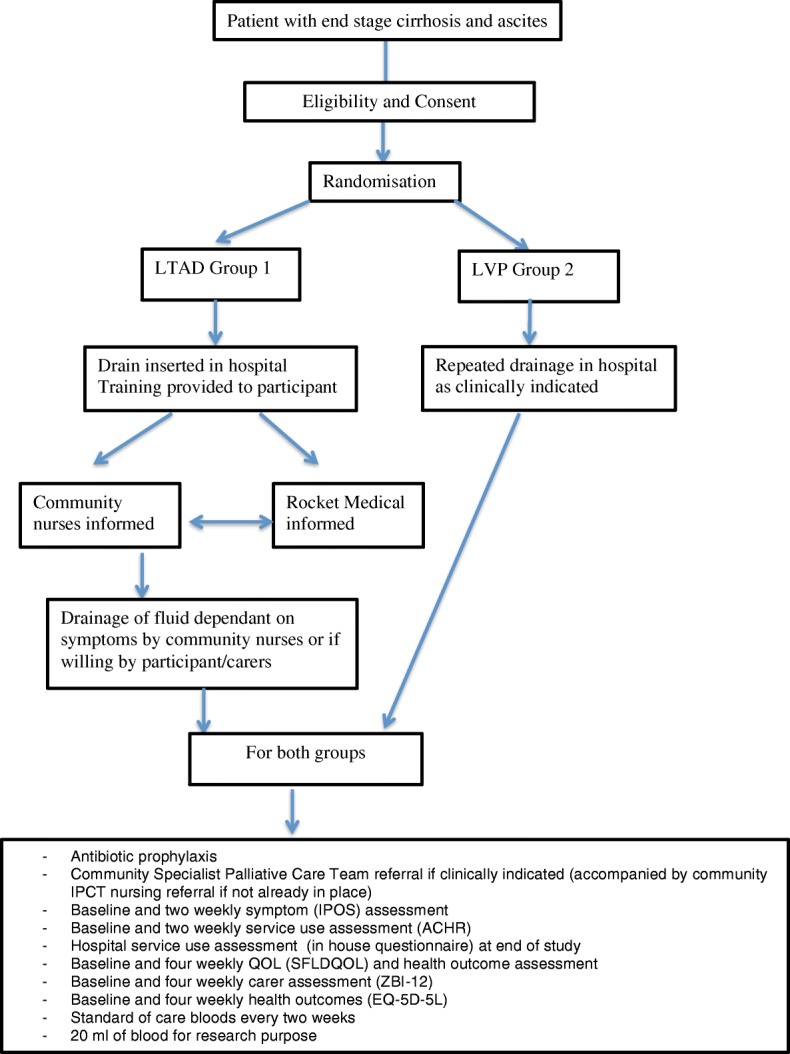
Participant timeline

Figure 2 shows the participant timeline/study flow chart.

### Schedule of assessments

**Fig. 3:**
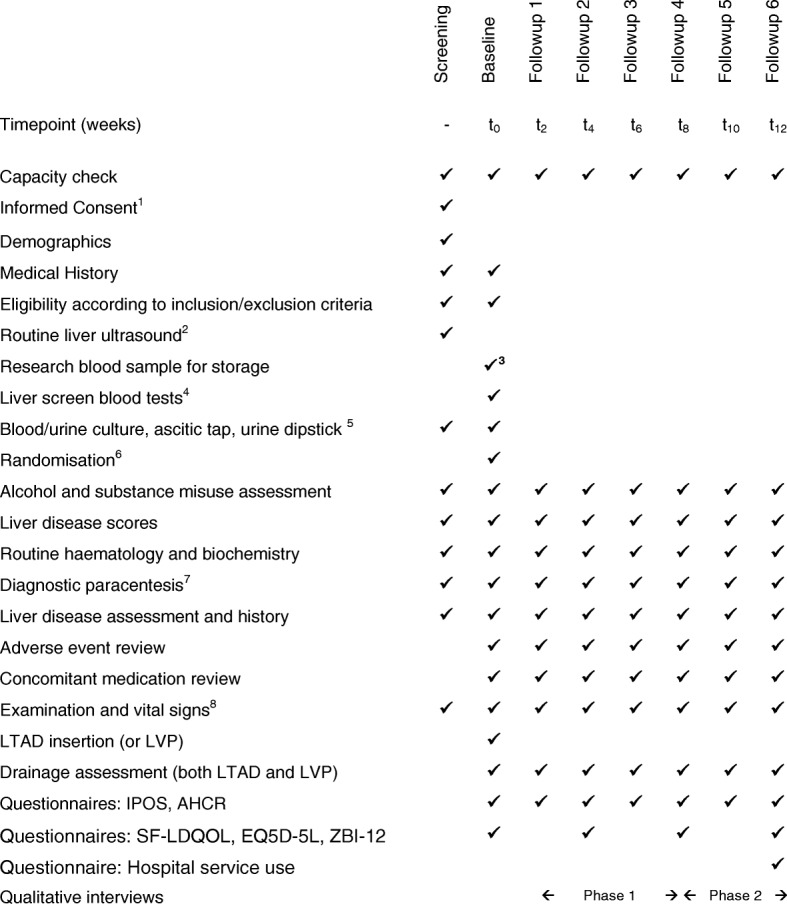
SPIRIT schedule

The research team member will visit participants at home every 2 weeks for questionnaire-based and clinical assessments (see the subsequent section) as well as collection of routine clinical blood samples. The amount and frequency of drainage and other pertinent observations will be recorded by community nurses in a formal study diary, as is the case when the LTAD is used in patients with ascites due to malignancy. The research team member will train and advise the community nurses and participants on data collection to reduce the possibility of missing data. See Fig. 2 and the Standard Protocol Items: Recommendations for Interventional Trials (SPIRIT) schedule (Fig. 3).

### Questionnaire-based assessments

The questionnaire-based assessments will be performed by the research team member and, depending on patient preference, will be done either face to face at the patient’s home or via telephone (within 3 days of the research team member visit). The research team member will follow guidance in the lone worker policy when conducting home/usual place of care visits.

We have selected questionnaires validated with our population group (e.g. palliative care patients and those with ESLD). Some, like the IPOS [28] (see the following section), are short, relatively brief to complete and have a proxy version if a patient loses capacity during the study. As this is a feasibility study, we will explore the acceptability of the measures used. We will pilot the patient questionnaires comprising the proposed measures with the first eight patients to explore and assess patient fatigue and time taken for completion. We will review the pilot findings and amend the patient questionnaire schedule if indicated, submitting the required Research Ethics Committee (REC) amendment for all proposed changes. The research team member will assist the participants in completion of the questionnaires if needed and if specifically requested by the participant. If participants are too unwell, the questionnaires can be completed by proxy by the carers, to reduce both the participant burden as well as the risk of missing data. Additionally, for those patients allocated to the LVP group, if the hospital visits coincide with questionnaire assessments, the assessments can be done at that point in hospital.

### Symptom distress and concerns

The IPOS [28–30] combines the Palliative care Outcome Scale (POS) and POS-S (POS with symptom list). These are measures frequently used in palliative care research and clinical practice [28–32]. They are validated for clinical practice, audit and research and can be used in any setting. The POS-S captures physical symptom specific information, and “other” symptoms specific to liver disease/ascites can be added, e.g. abdominal bloating. A SOP will be implemented when clinical and/or risk of harm issues are identified to ensure timely assessment by their usual healthcare providers and/or referral to a specialist palliative service depending on needs identified.

A staff version of the IPOS will be used in case participants are unable to complete the questionnaire. The IPOS will be assessed at baseline and two weekly and takes less than 10 min to complete (a total of 10 questions).

### Quality of life

QOL will be assessed using the Short Form Liver Disease Quality of Life (SF-LDQOL) [33, 34], a reliable and valid measure of health-related QOL in patients with advanced liver disease awaiting transplant, incorporating a core QOL assessment and disease targeted items. As specific QOL assessment tools are lacking in cirrhosis, the SF-LDQOL is the most appropriate option and was selected after service user involvement. The original SF-LDQOL questionnaire has 43 questions, though the questions from 26 onwards are for the purposes of validating the questionnaire and not specific to the SF-LDQOL itself [33, 34]. The authors have provided a scoring algorithm that includes only the first 25 questions; therefore, only these questions will be used. This questionnaire takes about 15 min to complete and will be assessed at baseline and four weekly.

### Health economics outcome

There are opposing views on the use of the EuroQol 5 dimensions (EQ-5D) as a composite measure of quality-adjusted life years (QALYs) in palliative care [18]. However, it is the most widely used indicator, and until valid alternatives are available, we have elected to assess the 5-level version (EQ-5D-5 L) [35] (at baseline and four weekly) in this feasibility study for its utility as an outcome. The EQ-5D-5 L has six questions and will take about 5 min to complete.

### Impact on carers

For those willing to participate, the Zarit Burden Interview (ZBI-12) [36, 37] will be employed at baseline and four weekly. The ZBI-12 measures family/informal carer appraisal of the impact of caregiving. It has 12 items, is easy to administer and it can be used in the hospital or community setting, taking about 10 min to complete.

### Service use assessment

For each arm of the feasibility study, a comprehensive patient-level database of services used will be collated, including all inpatient, outpatient, emergency, primary, community, social and voluntary services, equipment and supplies and assistance from family/informal carers. For community and home-based services, a modified version of the Ambulatory and Home Care Record (AHCR) [38] will be used and administered by the research team member at baseline and two weekly. The carers will assist with this, especially if the participant is too unwell. The AHCR, a standardised and comprehensive framework and tool, measures resources used within the end-of-life context from a societal perspective. This approach gives equal consideration to costs borne by the health system as well as those costs borne by care recipients and informal caregivers, such as family members and friends. It will take about 20 min to complete. Self-reported data will be verified and supplemented (e.g. for supplies) with reference to nursing records. Data on all hospital use will be gathered from hospital records at the end of the study using a purposefully designed in-house proforma. Service use will be converted to costs using national sources [39] and National Health Service (NHS) reference costs. Informal care will be valued using replacement cost methods and applying the tariff for community support workers.

A feasibility study gives us the opportunity to test candidate patient-reported outcome measures with the intention of only taking the most useful measures through to any definitive study. Survey fatigue was always a concern for the research team and our service users, and the issue also arose during the Ethics Committee review process. Therefore, we had a safety check to reassess survey fatigue after the first eight patients were recruited. We have found no evidence of any problems so far; indeed some patients restate their willingness to complete the questionnaires.

### Qualitative interviews

Qualitative data will be collected as part of a concurrent embedded strategy [40]. Interview themes will include an exploration of experiences of recruitment, participation, LTAD/LVP and end-of-life care; beliefs about the role and value of the LTAD in refractory ascites; and practical steps and barriers involved in undertaking the LTAD.

Twenty-eight optional interviews (with 20 participants and 8 clinical key informants) will be undertaken by a qualitative researcher, with additional support if needed. Clinicians and research participants will be identified and recruited via the research team member. Patient recruitment will seek to reach a maximum diversity sample of participants, i.e. interview participants with a wide range of demographic characteristics, with purposive sampling (if feasible), informed by the IPOS (given that the individuals of this cohort are living with deteriorating health). The research team member will invite patients to participate in the qualitative interview study and will seek permission to pass contact details (normally telephone number) to the qualitative researcher. The qualitative researcher will contact participants to arrange a convenient time for the interview. As life expectancy in refractory ascites due to ESLD is on average 6 months, the qualitative interview methodology seeks to explore a wide range of patient experiences, recognising that participant beliefs and experiences may change across this period. Interviews will, therefore, be divided into two phases:

- *Phase 1* (weeks 0–8) 12 patients (6 from each arm), 4 clinical staff
- *Phase 2* (weeks 9–12) 8 patients (4 from each arm), 4 clinical staff

In the event of inability to recruit new participants for Phase 2 interviews, additional participants or repeat interviews will be sought during Phase 1. Interviews with key clinical staff will follow the same aims of patient interviews and will be anonymous (i.e. key informants will be asked to withhold patient identities).

Interviews will take place at participants’ homes or by telephone according to participant preference and geographical location. Clinical staff will be interviewed at their place of work or at a mutually convenient venue. Consent will be taken from all participants, including any carer requested by the participant to be present. For telephone interviews, consent will be taken verbally and recording will be started before telephone consent is taken, so that the verbal consent can be recorded as a separate file from the interview. Signed consent forms will be kept for 5 years. Interview data will be transcribed and the audio version deleted. The anonymised transcription of the interview (including the verbal consent) will be stored (labelled with patient study number).

To reduce participant burden, breaks will be allowed during the interviews if requested by the participants, and interviews will last between 20 and 60 min.

### Safety monitoring

A monitoring plan will be put in place and adhered to for each research site (see Additional file 2). Monitoring visits will be undertaken by the Brighton and Sussex Clinical Trials Unit (BSCTU) on behalf of the Study Sponsor. The study may be audited in line with the BSCTU or by the Sponsor requirements. Audits will be conducted by personnel independent from the research team.

Common Terminology Criteria for Adverse Events (AEs) (CTCAE, version 4.03) [41] will be used when assessing AE and serious adverse events (SAEs). As this is a feasibility study, all AEs and SAEs will be recorded in the source data and reported on the eCRF. Crucially, only those SAEs that in the opinion of the CI are related to the study intervention (LTAD) will be reported in an expedited manner to the BSCTU.

This feasibility RCT is investigating the LTAD in a cohort with ESLD. By its very nature, this is a group with high morbidity and mortality. Hence, in this patient population, worsening of existing conditions, hospitalisations, acute illnesses and deaths are expected. These events will be recorded in the eCRF but will not be reported to the BSCTU or the REC.

Expected/unexpected unrelated AEs/SAEs will include but not be limited to:

- Hepatic encephalopathy
- Gastrointestinal bleeding related to peptic ulceration, hypertensive portal gastropathy or varices
- Liver cancer and or its treatment
- Complications of gastroscopy (perforation, bleeding)
- Complications of LVP (circulatory and or electrolyte disturbances, bleeding, bowel perforation, failed drainage)
- Complications of drug treatment for cirrhosis (lactulose, beta blockers, terlipressin, antibiotics, diuretics)
- Death related to the liver disease; will include death from liver failure, multiorgan failure, variceal bleeding and sepsis

### Expected serious adverse reactions (SARs)

If in the CI’s opinion a SAR is considered directly related to the LTAD and is an expected SAR, then this will be recorded on the eCRF and reported to the BSCTU immediately following the Safety Reporting SOP. Expected SARs will include the following (but only if they result in hospitalisation):

- Failure of LTAD insertion
- Drain leakage or blockage
- Cellulitis
- Bleeding
- Pain at site of insertion not controlled by analgesia
- Spontaneous bacterial peritonitis
- Sepsis which in the opinion of the CI is directly related to the LTAD
- Death which in the opinion of the CI is directly related to the LTAD

### Suspected unexpected, serious adverse reactions (SUSARs)

This category will include all SARs that in the opinion of the CI are directly related to the intervention and are not listed as a known (expected) SAR. All SUSARs that occur between insertion of the LTAD and 3 months post insertion or death, whichever is earlier, will be recorded on the eCRF and emailed/faxed to the BSCTU immediately, at least within 24 h of the research team becoming aware in accordance with the BSCTU Safety Reporting SOP. The REC will be notified of any SUSAR to the study intervention by the BSCTU. For each SUSAR, all relevant information will be collected, and the SUSAR will be followed up until resolved or a final outcome reached.

The CI will have direct and ultimate responsibility for reviewing all reported SARs and SUSARs and will ensure that the BSCTU reports these appropriately according to the BSUH SOP on Safety Reporting in Non-Clinical Trial of an Investigational Medicinal Product (CTIMP) studies.

### Data analysis

#### Statistical analysis

Guidelines for feasibility studies suggest that analysing 12 participants in each arm will provide an adequate sample size with which to achieve our objectives [42]. However, since this is a cohort with a poor prognosis, attrition is likely to be high. In our pilot study of seven patients [26], survival post insertion ranged from 6 to 96 days (though the LTADs were inserted late in the disease trajectory). Due to the advanced disease stage of the participants, we are assuming a 50% attrition. The sample size will therefore be increased to 24 participants in each arm, i.e. a total recruitment target of 48 participants. This sample size will be adequate to inform the research methods for a definitive phase 3 RCT.

Recruitment rate will be evaluated in terms of the proportion of eligible patients who provide informed consent. Attrition at all stages will be recorded, particularly due to unwillingness or inability to manage the LTAD, as this is an indication of acceptability. Data will be analysed on available cases in the groups to which they were randomised. We will present these data as a flow chart. The amount of missing data will be summarised for each variable, but there will be no imputation. As this is a feasibility study, stopping rules will not be defined.

**Fig. 4:**
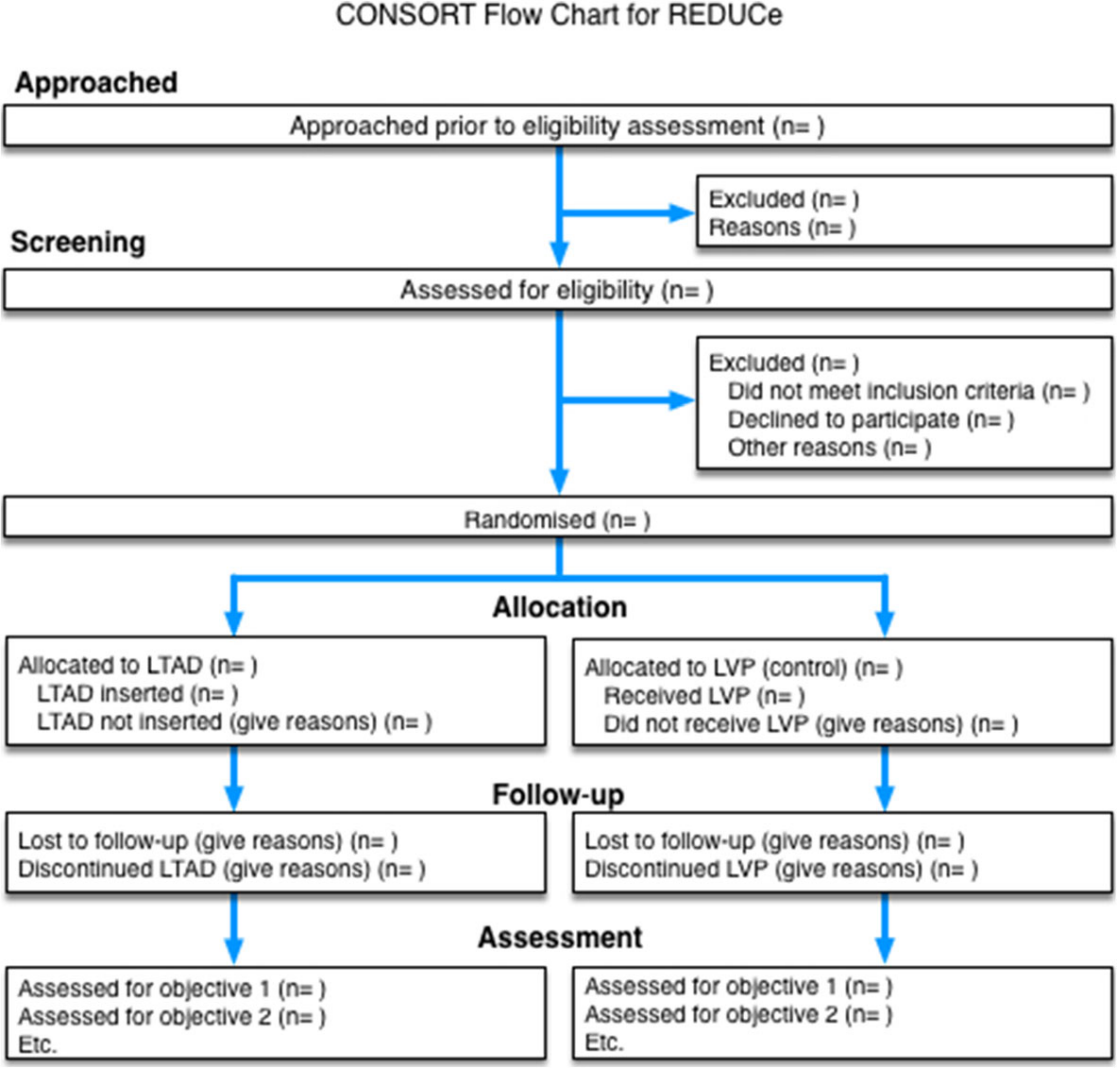
Consort flow chart

The flow of patients through the trial is depicted in the Consolidated Standards of Reporting Trials (CONSORT) diagram (Fig. 4) [43].

Descriptive statistics will be used to summarise and compare the quantitative outcome measures to include (1) complication rates: failed insertion, drain leakage or blockage, cellulitis, bleeding, pain at site of insertion not controlled by analgesia, peritonitis, sepsis and death (the latter two only if directly related to the LTAD), (2) symptoms: IPOS, QOL (SF-LDQOL, EQ-5D-5 L) [28, 33–35] and (3) carer burden [36, 37] for each arm. Means and standard deviations will be determined for normally distributed outcomes and medians and interquartile ranges for skewed outcomes at the different time points and at the end of the study. Analyses will use all available cases following intention-to-treat principles. We will calculate 95% confidence intervals for parameter estimates as appropriate. Prior to the analysis of the data, a detailed statistical analysis plan will be written and signed off.

#### Health economics data analysis

The economic analysis will adopt the perspectives of the health and social care systems.

Using the patient-level database assembled from participant self-report and hospital and community nursing records, the feasibility study will identify the main resource items for which comprehensive data collection would be required in the main trial. Interactions between ascites management and other palliative care services will be explored. In particular, community nurse visits in both groups will be monitored, so *extra* visits required for the LTAD, compared to normal care, which was a major source of uncertainty in the earlier modelling study, can be identified [13]. The group mean total costs of services used in ascites management will be compared between LTAD and LVP.

The properties of the main clinical outcomes (IPOS, SF-LDQOL, EQ-5D-5 L) [28, 33–35] and the number of hospital days will be investigated to assess their value as measures of effectiveness for the definitive trial so that a primary outcome can be determined. Data on QALYs from EQ-5D-5 L [35] will be investigated for possible use in the economic evaluation. A preliminary cost-effectiveness analysis will be undertaken to determine the likely advantage of conducting a full trial [44]. A sensitivity analysis will be performed by varying the key cost drivers, such as the number of inpatient days and the cost of bed days.

#### Qualitative data analysis

If purposive sampling is not feasible, the proportion of participants choosing to participate in qualitative interviews will be noted. Interviews will be audio recorded and labelled using the same anonymous study number as the intervention component of this study. The same number will be used so that in the event of a participant reporting a serious concern about his/her condition or care, the qualitative researcher can raise this concern with the patient’s clinician. In this unlikely event, the researcher will inform the patient of the need to convey this information to the clinician. The qualitative researcher will have access to the clinical study data of the individual if needed.

Thematic analysis supported by qualitative software (NVivo) [45] will be used to extract overarching themes from the interviews to capture patients’ experiences and beliefs. Utilising the process of triangulation [46], the findings of the qualitative arm will be used to inform the quantitative results, particularly in the context of QOL and experience of end-of-life care provision.

Data will be analysed in a blinded manner. However, the research team members collecting information from the patients will always be aware of their allocation since a high level of scrutiny is necessary to ensure that there are no safety events in the LTAD group. Our service users were also insistent that participants not engage with multiple members of the research team, further excluding blinded data collection.

### Ancillary and post-trial care

At the end of the trial, participants will continue to be assessed by their usual medical care team. Those allocated to the LTAD arm will have the option, if they so wish, to continue with the LTAD under care of their usual consultant gastroenterologist/hepatologist.

### Committees

#### Trial Management Group (TMG)

The TMG will comprise the CI, all co-investigators and PIs, research fellow, trial manager, data manager and statistician and will be chaired by the CI (SV).

The TMG will meet every month to:

1. Finalise trial-related materials
2. Oversee and co-ordinate the various aspects of the project, so that the research completes on time and on budget
3. Assess study progress to ensure that recruitment is on target and on budget. If recruitment is below that anticipated, then strategies to improve this will be discussed
4. Assess adherence to protocol by reviewing protocol deviation logs.

#### Data Safety Monitoring Committee (DSMC)

The DSMC will be an independent committee chaired by Professor Guruprasad Aithal (Professor of Hepatology, Nottingham University NHS trust) with two other independent members, Professor Bobbie Farsides (Chair of Medical Ethics at BSMS) and Professor Martin Llewelyn (Professor in Infectious Diseases, BSMS and Hon Consultant, BSUHT), as well as at least one service user member. A study data report will be provided to the DSMC by the trial manager every 10–12 months for the first 2 years and then every 6 months for the last year, in accordance with the Terms of Reference for the Committee. The DSMC will meet as above to address any safety concerns, review any ethical issues raised and monitor adverse events. The DSMC will make recommendations to the TMG as appropriate and has the power to stop the trial if necessary. The DSMC will be independent of the Study Sponsor. Details of the Independent Data Monitoring Committee (IDMC) charter can be obtained from trial.monitors@bsuh.nhs.uk.

## Discussion

The impetus for the REduced Drainage Utreatable Cirrhosis (REDUCe) trial was driven by our concerns that patients with ESLD receive suboptimal end-of-life care compared to those with other terminal conditions. Most individuals with ESLD almost always die in hospital [6] while receiving end-of-life care, even though in many cases palliative care provided within the community would be more appropriate and compassionate. Such an option is often not feasible due to the complex end-of-life needs of patients with ESLD (including LVP) and the fluctuating disease trajectory making it difficult to define when a palliative phase has been reached [6, 47]. Most patients with ESLD develop ascites [2, 3], and the management strategy in ESLD is thus often dictated by this specific symptom. Our own data suggest that approximately 40% of patients with ascites requiring LVP can go on to develop refractory ascites [48]. This condition has a major impact on the QOL in ESLD, due to factors such as direct physical discomfort but also the need for recurrent hospitalisation for LVP [3, 49]. We would argue that for many such individuals these recurrent hospitalisations impair their QOL. A more appropriate option would be to focus on holistic palliative care in the community, based on discussions on future wishes [49, 50]. This will require a multidisciplinary approach to the disease, reflected in the composition of the REDUCe study team.

Consistent with the lack of research in this complex cohort of individuals and therefore not unexpectedly, recruitment to this trial has been challenging. We found that clinicians who were not part of the study team were often reluctant, particularly in younger patients, to diagnose ESLD and discuss the implications of a limited life expectancy and purely palliative management. In some instances prospective participants were only identified late and unfortunately died before they could make an informed decision about trial participation. The patients themselves are often vulnerable and (in their opinion) disenfranchised and stigmatised. Finally, setting up new sites for a study that spans both acute hospital and community settings, often without existing research collaborations, has been difficult and time-consuming.

Conversely, we have already begun to note positive changes in attitudes, beliefs and practice of HCP locally and in the study sites. Simply by attempting to discuss the REDUCe trial, we have seen a change in attitudes towards symptom control and QOL as well as timely referral to palliative care. There is also increasing recognition that patients should be able to be more involved in decisions about their end-of-life care. This trial has raised the local profile of these under-researched patients, with wider recognition of the need for MDT communication and collaboration. Specifically, many local hospitals now discuss all patients with ESLD at a weekly liver MDM so as to identify in a timely manner those who are entering the palliative phase of their disease. This has undoubtedly driven the improvement in recruitment. The National Institute for Health Research (NIHR), acknowledging this and the potential of this study to result in a paradigm shift in end-of-life care in ESLD, has granted a funded extension for a year.

### Trial status

The trial is now open, with 36 patients recruited as of May 2018. Funded extension was obtained May 2017.

## Additional files

Additional file 1: REDUCe Study. (PDF 69 kb)

Additional file 2: Monitoring plan. (PDF 395 kb)

## Abbreviations

AE: Adverse event
AHCR: Ambulatory and Home Care Record
ALFA: Automated low-flow ascites
AMA: Antimitochondrial antibody
ANA: Antinuclear antibody
BSCTU: Brighton and Sussex Clinical Trials Unit
BSMS: Brighton and Sussex Medical School
BSUH: Brighton and Sussex University Hospital
CI: Chief Investigator
CLD: Chronic liver disease
CTIMP: Clinical Trial of an Investigational Medicinal Product
eCRF: Electronic case report form
ESLD: End-stage liver disease
GCP: Good Clinical Practice
GP: General practitioner
HBsAg: Hepatitis B surface antigen
HCP: Healthcare professional
HCV: Hepatitis C virus
HIV: Human immunodeficiency virus
HRA: Health Research Authority
INR: International normalised ratio
IPCT: Integrated Primary Care Team
IPOS: Integrated Palliative care Outcome Scale
KCL: King’s College London
KCTU: King’s College London Clinical Trials Unit
LKM: Liver kidney microsomal antibody
LTAD: Long-term abdominal drain
LVP: Large volume paracentesis
MDM: Multidisciplinary meeting
MDT: Multidisciplinary team
MRC: Medical Research Council
NHS: National Health Service
NIHR: National Institute for Health Research
PI: Principal Investigator
PIS: Patient information sheet
POS: Palliative Outcome Score
POS-S: Palliative Outcome Score (Symptom list)
PRH: Princess Royal Hospital
QALY: Quality-adjusted life year
QOL: Quality of life
R&D: Research & development
REC: Research Ethics Committee
RfPB: Research for Patient Benefit
SAE: Serious adverse event
SAR: Serious adverse reaction
SF-LDQOL: Short Form Liver Disease Quality of Life
SMA: Smooth muscle antibody
SOP: Standard operating procedure
TIPS: Transjugular intrahepatic portosystemic shunt
WSHFT: West Sussex Hospital Foundation Trust
ZBI-12: Zarit Burden Interview
