# Correction to: Palliative long-term abdominal drains versus repeated drainage in individuals with untreatable ascites due to advanced cirrhosis: study protocol for a feasibility randomised controlled trial

**DOI:** 10.1186/s13063-018-2883-1

**Published:** 2018-09-13

**Authors:** Lucia Macken, Louise Mason, Catherine Evans, Heather Gage, Jake Jordan, Mark Austin, Nick Parnell, Max Cooper, Shani Steer, Justine Boles, Stephen Bremner, Debbie Lambert, David Crook, Gemma Earl, Jean Timeyin, Sumita Verma

**Affiliations:** 1 Department of Clinical and Experimental Medicine, Brighton and Sussex Medical School, University of Sussex, Main Teaching Building, North South Road, Falmer, Brighton, East Sussex, BN1 9PX UK; 2 Department of Gastroenterology and Hepatology, Brighton and Sussex University Hospitals Trust, Royal Sussex County Hospital, Eastern Rd, Brighton, East Sussex, BN2 5BE UK; 3 Department of Palliative Medicine, Brighton and Sussex University Hospitals Trust, Royal Sussex County Hospital, Eastern Rd, Brighton, East Sussex, BN2 5BE UK; 4 King’s College, Cicely Saunders Institute, Department of Palliative Care, Policy and Rehabilitation, Bessemer Road, London, SE5 9PJ UK; 5 Sussex Community NHS Foundation Trust, Brighton General Hospital, Elm Grove, Brighton, BN2 3EW UK; 6 Surrey Health Economics Centre, School of Economics, Faculty of Arts and Social Sciences, University of Surrey, Guildford, Surrey, GU2 7XH UK; 7 Brighton & Sussex Clinical Trials Unit, University of Brighton, Room 204 Bevendean House, Falmer, BN1 9PH UK; 8 Department of Primary Care and Public Health, Brighton and Sussex Medical School, Mayfield House, Brighton, BN1 9PH UK

## Correction

Following publication of the original article [1], the authors reported that the figure legend for Figure 3 was absent. In addition, they have requested additional funding information to be added. In this Correction the initial and updated funding information are shown. The original publication of this article has been corrected.

Initial funding information:

Funding was provided by the UK NIHR Research for Patient Benefit scheme (reference PB-PG-0214-33068). LMa has also received additional funding from Kent Surrey and Sussex Deanery. CE is funded by the Health Education England (HEE)/NIHR Senior Clinical Lectureship.

Updated funding information:

This manuscript presents independent research funded by the NIHR under its Research for Patient Benefit (RfPB) Programme (Grant Reference Number PB-PG-0214-33068). The views expressed are those of the authors and not necessarily those of the NHS, the NIHR or the Department of Health.

LMa has also received additional funding from Kent Surrey and Sussex Deanery. CE is funded by the Health Education England (HEE)/NIHR Senior Clinical Lectureship.

The legend for Figure 3 is as follows:

1. Informed consent can be given prior to the screening visit, but must be confirmed at the screening visit.
2. Unless previous imaging (CT, MRI or ultrasound) is available within the previous six months.
3. 20 ml of blood will be taken at baseline for future ethically approved research, (10 ml saved as serum, and 10 ml as whole blood as per laboratory SOP).
4. HBsAg, HCV antibody, HIV antibody, ANA (antinuclear antibody), AMA (Antimitochondrial anti-bodies), SMA (smooth muscle antibody), LKM (kidney microsomal antibody), serum ferritin, serum copper, serum caeruloplasmin, serum alpha-1 antitrypsin, fasting serum total cholesterol, triglycerides, high density lipoprotein (HDL) cholesterol, total cholesterol:HDL ratio and low density lipoprotein (LDL) cholesterol (if not done in the previous three months).
5. If not performed within the last 48 h or 14 days prior to baseline visit.
6. Randomisation can be done up to 14 days prior to the baseline visit.
7. If not performed within the last two (screening visit) or seven days (baseline visit). For subsequent visits this will be done if clinically indicated (Group 1: LTAD) or, in the case of Group 2 (LVP), at each visit.
8. Temperature, blood pressure and pulse; height and weight at baseline visit only.
