# Supplementary material for: Palliative long-term abdominal drains versus repeated drainage in individuals with untreatable ascites due to advanced cirrhosis: study protocol for a feasibility randomised controlled trial
Source: Trials. 2018 Jul 27;19:401. doi: 10.1186/s13063-018-2779-0 (PMC6062920; doi:10.1186/s13063-018-2779-0)
Supplement: Supplementary file 1 — REDUCe Study. (PDF 69 kb) [file 13063_2018_2779_MOESM1_ESM.pdf]

REDUCe Study

Title of study: Palliative long-term abdominal drains versus repeated drainage in individuals with untreatable ascites due to advanced cirrhosis: a feasibility randomised controlled trial

ASSESSING CAPACITY TO CONSENT- CHECKLIST FOR RESEARCHERS RECEIVING CONSENT FOR THE REDUCe Study

At ***this specific time*** of gaining consent for this person is there any evidence that:

- The person does **not** have a general understanding of what decision they need to make and why they need to make it?
- The person does **not** have a general understanding of the likely consequences of making this decision?
- The person is **unable** to understand, retain, use or weigh up the information relevant to this decision?
