# Supplementary material for: Palliative long-term abdominal drains versus repeated drainage in individuals with untreatable ascites due to advanced cirrhosis: study protocol for a feasibility randomised controlled trial
Source: Trials. 2018 Jul 27;19:401. doi: 10.1186/s13063-018-2779-0 (PMC6062920; doi:10.1186/s13063-018-2779-0)
Supplement: Supplementary file 2 — Monitoring plan. (PDF 395 kb) [file 13063_2018_2779_MOESM2_ESM.pdf]

Monitoring Plan

**Study Title**: REDUCE: Palliative Long-term Abdominal Drains versus Repeated Drainage for Ascites due to Cirrhosis	
**Sponsor Reference**: 173423	**EUDRACT Number**: n/a
**REC reference:** 15/SC/0257	
**Chief Investigator:** Dr Sumita Verma	
**Sponsor:** Brighton and Sussex University Hospital NHS Trust	

Primary Objective (P)

Acceptability of LTAD to patients/carers/ staff and whether the preliminary cost-effectiveness analysis indicates that LTAD is potentially cost-effective without affecting the patient/ carer experience, or if LTAD is more costly than LVP but preferred by service users.

Specifically whether:

a. Percentage of study period time in hospital for LTAD group is < 50% of that for the LVP group (where the study period time is the number of days from randomisation to the end of the study period or the patient's death (whichever is earliest); time spent in hospital is the number of bed days used)

b. Attrition rate is not > 50%

c. There is <10% overall rate of LTAD removal due to one or more of the following complications: peritonitis, failed insertion, bleeding and blockage

d. 80% of questionnaires and qualitative interviews completed by each patient.

Secondary Objectives (S):

n/a

SUMMARY OF MONITORING ARRANGMENTS

CTUSOP003 'Monitoring' will be followed by the trial monitor.

Type of Monitoring:

Central monitoring will be carried out.

Site Initiation Visit:

The SIV should involve a face to face or online meeting with the CI, Research Fellow, Trial Manager, monitor, research team and any other relevant supporting services. Relevant research staff from the research site that should be present include the PI, co-Investigators, research nurses, research lab staff and community nurses.

Attendance at this training should be documented on the Site Initiation Training Log. Any further study training provided to research staff after this session must be documented on a Training Log.

A Site Initiation Report will be produced by the Trial Manager and sent to the CI and all of the relevant research staff within 10 working days of the SIV. This will detail any outstanding issues and assign responsibilities for follow up before the site can be activated.

When the site is ready to begin recruitment, the site will be sent a 'Site Activation' notification by the Trial Manager.

Minimum documents required by the coordinating centre prior to 'Site Activation' notification are as follows:

- Copy of completed delegation log
- Copy of completed SIV training log and any other training logs/evidence of training for those who were unable to attend the SIV
- Copies of CVs, GCP certificates and honorary contracts (if applicable) for all those on the delegation log
- Locally headed PIS, ICF and GP letter and any additional documents
- Copy of local R&D confirmation of capability and capacity
- Copy of local contract
- Copy of the Monitoring Plan signed by PI
- Details of archiving arrangements at local site
- eCRF training for data entry staff

Central Monitoring:

- Central verification checks on the eCRF will be performed throughout the study by the Trial Manager or delegate, in accordance with the Data Management Plan.

Site specific monitoring

Item	Relevant document	Checks to complete	Frequency
ISF completeness	ISF checklist	Check that no documents listed are missing	every 6 months
Staff training and responsibilities	Delegation log	Confirm that randomisation is being carried out by delegated person	every 6 months
Training records	CV	All staff signed off by PI and trained	every 6 months
	GCP	Protocol training, GCP, CV are up to date	every 6 months
	Protocol training log		every 6 months
Samples storage	Sample storage records	Send copy of sample storage spreadsheet	every 6 months
Screening/enrolment of participants	Screening and enrolment log for participants	Confirm all participants are listed, log fully complete, review refusal or ineligibility	every 6 months
Enrolment log of carers	Screening and enrolment log for carers	Confirm log fully complete	every 6 months
		Cross check that questionnaires completed for all carers enrolled on MACRO	every 6 months
Worksheets		Cross check that any changes to the protocol have been reflected in worksheets if required	every 6 months

Participant specific monitoring			
**Item**	**Relevant document**	**Checks to complete**	**Frequency**
Eligibility checks	Eligibility form<br>Liver Disease History<br>H&B<br>Ascitic tap<br>Urine<br>Blood culture	Complete MACRO checks from Data Validation Plan<br>Child Pugh<br>Liver disease score form<br>H&B: Bilirubin q8a, Albumin q14a, INR q7,<br>Ascites<br>Encephalopathy<br>Infection: check H&B, Ascitic tap, urine, blood culture<br>Liver Disease History: reason not for transplant<br>Prior hepatic encephalopathy	Ongoing (Immediately after each randomised patient)
Randomisation	Randomisation list from MACRO	Cross check MACRO randomisation list with MACRO treatment	every 6 months
Adverse event/conmed review	MACRO checks from DVP	Check that no SAEs missed from reporting.	every 6 months
		Cross check conmeds with AEs listed	every 6 months

		Check that all AEs resolved/reviewed	every 6 months
Deaths	Cross check MACRO with Data Patient Status log	Check deaths have been reported correctly - withdrawal form completed. Withdrawal log	every 6 months
Drains and drainage bags logs	REDUCE Rocket Medical Drains and Bags Log- v1.0 - 11 Oct 2015	Check total number provided per patient versus total agreed to be provided per patient	every 6 months
Data checks	Automated checks - review outstanding queries<br>Data Validation Plan - manual queries<br>Review missing data	Complete checks	every 6 months - when a patient completes treatment

Qualitative study			
Participant details	Qualitative screening/enrolment log	Check screening/ enrolment log fully completed	every 6 months
Consent	Consent form	Check completed appropriately by Qualitative Researcher	every 6 months
		Verbal consent recorded and stored in secure backed up area	every 6 months
		Interview separately recorded and stored in secure backed up area (check start and end of recording)	every 6 months

- Site Data Officers can perform missing data checks and the TM/DM will regularly follow up to ensure completeness of data entry.
- A DMC will meet approximately every six months to:
    - address any safety concerns
    - review any ethical issues raised
    - • monitor adverse events
- A Central Monitoring Report will be completed.

On Site Monitoring Visits:

No onsite visits are planned, however, if issues are identified by the Trial Manager as a result of central monitoring, then onsite monitoring visits may be required.

Close Out Monitoring

All outstanding queries must be resolved or confirmed as unresolvable<br><br>The ISF must be complete and final ISF checklist returned to BSCTU by the site.<br><br>The completed protocol deviations list must be reviewed, signed and sent to the trial statistician.<br><br>An official close out letter will be sent by the trial manager to each participating site.<br><br>The TMF must be reviewed and confirmed as complete, prior to archiving.

This plan will be kept under review and modified as necessary throughout the duration of the trial.

Senior Trial Manager: On behalf of Sponsor

Print: ______________________

Sign: ______________________

Date: ______________________

Chief/Principal Investigator:

Site: PLYMOUTH HOSPITALS NHS TRUST

Print: DR DAVID SHERIDAN

Sign: [signature]

Date: 02 FEB 2017
